# A comparative study of positive and negative electronic word-of-mouth on the SERVQUAL scale during the COVID-19 epidemic - taking a regional teaching hospital in Taiwan as an example

**DOI:** 10.1186/s12913-022-08930-2

**Published:** 2022-12-22

**Authors:** Po-Chun Lee, Li-Lin Liang, Min-Hsin Huang, Ching-Yuan Huang

**Affiliations:** 1 Department of Internal Medicine, Kaohsiung Armed Forces General Hospital, Kaohsiung, Taiwan; 2 Department of Business Management, National Sun Yat-sen University, Kaohsiung, Taiwan; 3 Institute of Public Health, National Yang-Ming Chiao Tung University, Taipei, Taiwan; 4 Department of Marketing Management, SHU-TE University, Kaohsiung, Taiwan

**Keywords:** Electronic WOM (eWOM), SERVQUAL, Quality of medical service, COVID-19 pandemic, Regional teaching hospital

## Abstract

**Background:**

In recent years, studies have shown that electronic WOM (eWOM) directly reflects consumers’ post-purchase psychological perception and directly affects repurchase behavior. This information is valued by institutions in various fields. Within the scope of the evaluation of service characteristics, medical service is the least visible and most difficult service attribute to evaluate. Service organizations must have high trust attributes. Therefore, an eWOM review significantly influences people’s decision-making process when choosing a healthcare provider. The purpose of this research is to combine eWOM reviews with the SERVQUAL scale in a comparative study of positive and negative eWOM reviews of a regional teaching hospital in Taiwan.

**Methods:**

This research obtained data from publicly available eWOM reviews on Google Maps of a regional teaching hospital in Taiwan over the past 10 years (from June 24, 2011, to December 31, 2021) using website scraping technology. The semantic content analysis method was used in this study to classify eWOM reviews according to the revised PZB SERVQUAL scale.

**Results:**

Statistical analysis was conducted. During the COVID-19 pandemic, positive reviews showed a downward trend. Among the five determinants of the SERVQUAL of PZB, positive eWOM reviews performed best in “assurance” with a positive review rate of 60.00%, followed by 42.11% for “reliability”. For negative eWOM reviews, “assurance” performed the worst with a positive rate of 72.34%, followed by “responsiveness” at 28.37% and “reliability” at 26.95%.

**Conclusion:**

Since the onset of COVID-19 in 2020, negative eWOM has increased significantly and exceeded the amount of positive eWOM. Regardless of positive and negative reviews, what patients care most about is “assurance” of the professional attitude and skills of medical staff, which urgently needs to be strengthened. In addition, good “reliability” will help to develop positive eWOM. However, “responsiveness” as indicated by poor service waiting time can easily lead to the spread of negative eWOM. Hospital management should focus on these service-oriented qualities.

## Introduction

During the epidemic, as hospitals became battlegrounds in the fight against COVID-19, nonemergency medical services were suspended and medical resources were tightened to prevent further increases in the overall workload of hospitals. Fear of contracting COVID-19 has changed interactions with healthcare professionals and hospitals. Patients are afraid to seek medical attention because hospitals are seen as places with a high risk of COVID-19 transmission [1]. As a result, patients delay medical care and miss the prime treatment time [2, 3]. In addition to fear of the known, people are also afraid of the unknown due to COVID-19 [4]. The fear of the spread of COVID-19 has been heavily publicized by traditional and social media. For these reasons, in most areas, people have turned to use websites to search for information on the reputation of hospital services when choosing medical treatment during the epidemic.

In the current era of online media, electronic word-of-mouth (eWOM) spreads like viral marketing, and it spreads faster and wider than traditional word-of-mouth [5–7]. eWOM and online reviews are the best channels for consumers to instantly respond with postconsumer psychological cognition about service organization communication and repurchase intentions [8–12]. The research results of many previous studies have shown that the long-term impact of the word-of-mouth effect of online reviews is far greater than that of traditional marketing activities and media exposure [13–18]. In recent years, research on eWOM and online reviews in the field of business management has received considerable attention [19–23]. However, although the valence of online reviews is mostly positive, the influence of negative reviews is stronger than that of positive reviews, and there is a phenomenon of negative bias [24–27].

This study was motivated by a review of the previous eWOM research literature, which has mainly focused on catering and tourism services and e-commerce services, while studies of eWOM reviews of medical institutions are lacking [7, 23, 26, 28–30].

In the evaluation spectrum of Zeithaml’s service characteristics, medical services are the least tangible and the most difficult service benefits to evaluate [31, 32]. Medical service organizations must have high trust attributes. Therefore, eWOM reviews significantly impact people’s decision-making process when choosing medical institutions. In the past few years, service management research on medical institutions has mostly focused on the discussion of service quality. Commonly used approaches are the PZB (Parasuraman, Zeithaml, and Berry) service quality gap model and the SERVQUAL (Service Quality) scale [33–43]. From the comprehensive literature review, it can be found that SERVQUAL has good reliability and validity as well as wide applicability [35, 37, 39, 40, 44–47]. The aim is to identify broad areas of company service quality deficiencies and strengths as a diagnostic method, but also has applicability in different cultural contexts [48]. There are also many scholars through the literature aggregation studies to confirm that “SERVQUAL” is one of the most commonly used models to measure the quality of medical services [48–53]. Previous PZB studies used questionnaires to obtain primary data on subjects’ responses to service quality and have rarely used secondary eWOM data for classification and discussion [54]. eWOM is the most authentic and direct response from customers at the moment they are being served. Questionnaire surveys generally have the shortcoming of retrospective memory and the limitation of deliberate concealment.

According to practical observations, Google mapping in Taiwan is not for profit, which is more objective than word-of-mouth on general social networking sites. Most Taiwanese hospitals do not manage comments on Google mapping, which has become a loophole in hospital service management. Moreover, Taiwan’s medical institutions prohibit sales-oriented business practices (Article 61 of the Taiwan Medical Law), so they cannot distort the content of electronic reviews.

This study aims to use the eWOM of real hospital customer responses to replace the previous SERVQUAL questionnaire survey of hospital customers’ responses to service quality, which can more accurately reflect the hospital’s service quality and satisfaction. The results of this study are analyzed by data mining techniques using the positive and negative eWOM of hospital customers to evaluate the performance of SERVQUAL in five dimensions to collect the feelings of hospital customers during the service process. This can specifically reflect the service of medical institutions, which is the main factor for the improvement of the project [55–57].

This study sets the following topics:

1. To explore the development trend of positive and negative eWOM reviews before and after a single hospital outbreak;
2. To explore whether the word length of positive and negative eWOM comments in a single hospital affects the score;
3. To explore the presence status of the positive and negative eWOM comments in a single hospital in the five dimensions of SERVQUAL.

## Methods

This study adopted the SERVQUAL scale proposed by PZB as the theoretical basis of medical service quality [37, 39–43, 58, 59]. The five dimensions of the PZB SERVQUAL scale are defined as follows: “Tangibles: Physical facilities, equipment, and appearance of personnel; Reliability: Ability to perform the promised service dependably and accurately; Responsiveness: Willingness to help customers and provide prompt service; Assurance: Knowledge and courtesy of employees and their ability to inspire trust and confidence; Empathy: Caring, individualized attention the firm provides its customers” [38].

This study makes minor revisions to the SERVQUAL scale designed by Lee for patients in the Taiwan National Army Hospital to facilitate the classification and statistical analysis of eWOM reviews [38]. This study and the previous SERVQUAL scale explored the largest differences in the quality of medical services, but most previous studies used the SERVQUAL scale to design questionnaires to conduct quantitative tests. The current study is based on eWOM reviews extracted through website scraping technology for semantic content analysis that were categorized into the revised SERVQUAL scale and then statistically analyzed. This study used web scraping techniques written in the Python programming language [55–57]. The content of word-of-mouth reviews published by Google Maps of the research target organization was extracted, and these review data were formatted into a more convenient Excel sheet and then classified and analyzed based on the modified PZB SERVQUAL scale using semantic content analysis. The length of the review affects the rating; the longer a positive review is, the higher the score, while the longer a negative review is, the lower the score [5, 60–63]. The coding procedure was conducted by the fourth author of this study (a doctor of management with expertise in service industry management and familiarity with the application of the PZB-SERVQUAL five-dimensional scale). The second author (a doctor of management) and the experts for this study (Ph.D. in Management) performed a “recheck after coding” to ensure the reliability and consistency of the data and then conducted a statistical analysis. It is possible to review the factors of service success and service failure of medical institutions in a more specific manner and provide more specific solutions to confirm service quality problems and improve service quality. Therefore, this study has both qualitative and quantitative research value. This research obtained 430 eWOM and online reviews on Google Maps from a regional teaching hospital in Taiwan from June 24, 2011, to December 31, 2021, through website scraping technology. After the screening, 38 reviews that were not relevant to service quality were eliminated; thus, only 221 valid positive service reviews and 171 valid negative eWOM reviews were obtained. The semantic content analysis method was used to classify the reviews according to the revised PZB SERVQUAL scale 6, and SPSS 20 statistical application software was used for statistical analysis.

## Results

### Trend analysis of positive and negative customer reviews over time

**Fig. 1:**
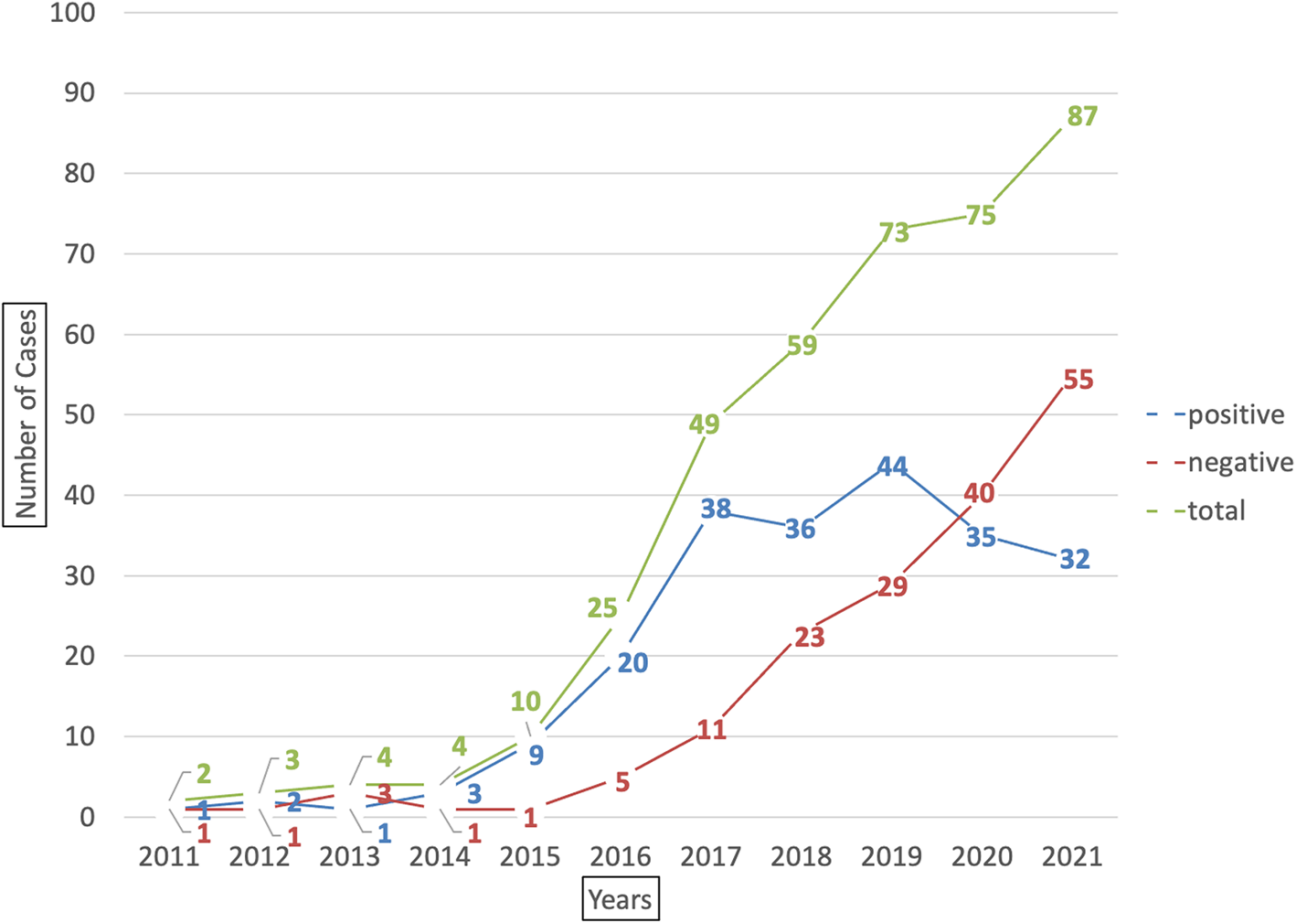
Trends in the number of positive and negative eWOM reviews over the years. Data collection period 2011/06/24~2021/12/31

According to Fig. 1, the hospital’s total number of eWOM reviews has increased over the years. Since 2015, eWOM reviews have risen sharply. Positive eWOM growth showed a downward trend beginning in 2019, while negative eWOM continued to rise. Since the onset of COVID-19 in 2020, negative eWOM has increased significantly and exceeded the amount of positive eWOM. The hospital’s negative reputation during the pandemic was due to the government’s health and welfare policy, and uncontrollable medical factors caused more dissatisfaction and negligence in services. This study used an independent-samples t-test to test the difference between the numbers and scores of positive and negative reviews before and after the COVID-19 outbreak. Table 1 shows that there was a difference in positive review scores before and after the COVID-19 outbreak (*P* < 0.003**). Scores during the outbreak (mean = 4.72) were higher than those before the outbreak (mean = 4.56). During the epidemic, patients provided positive eWOM for the medical treatment they received.

**Table 1:** Differences between positive and negative review scores before and after the epidemic

Dependent variables	Before and after the outbreak	N	AVG	The F Value	Sig.
Positive review score	Before outbreak	154	4.56	8.810	0.003**
	After outbreak	67	4.72		
Negative review score	Before outbreak	76	1.43	19.938	0.000***
	After outbreak	95	1.19		

The time before and after the epidemic is defined as “severe special infectious pneumonia” (COVID-19) as the fifth notifiable infectious disease announced by Taiwan’s Ministry of Health and Welfare on 15 January 2020

*N*  Number, *AVG*  Average, *Sig*  Significance

*P*<0.001

During the epidemic, the number of negative reviews after the outbreak was greater than the number before the outbreak, and there was a significant difference in the negative review scores (*P* < 0.001***). Post-outbreak negative scores (mean = 1.19) were lower than pre-outbreak negative scores (mean = 1.43). The dissatisfaction of patients with the medical treatment process during the epidemic affected the service reputation.

### Comparative statistical analysis of positive and negative scores

According to Table 2, the positive eWOM score was 5 points, or 67.87%. According to Table 3, the negative eWOM score was 1 point, or 79.53%. Therefore, it can be inferred that patients are motivated to leave comments online when they are highly satisfied or extremely dissatisfied.

**Table 2:** Statistical analysis of positive WOM reviews

Score	Number	Number (%)
3 points	16	7.24%
4 points	55	24.89%
5 points	150	67.87%
Total	221	100.00%

The scoring range is 1 to 5 points; 3 to 5 points are classified as positive; 3 points for reviews are classified as positive or negative based on the meaning of the message

**Table 3:** Statistical analysis of negative WOM reviews

Score	Number of times	Number of times (%)
1 point	136	79.53%
2 points	19	11.11%
3 points	16	9.36%
Total	171	100.00%

The scoring range is 1 to 5 points; 1 point and 2 points for no reviews are classified as negative; 3 to 5 points are classified as positive; 3 points for reviews are classified as positive or negative based on the meaning of the message

### Comparative analysis of the importance of positive and negative reviews

According to Table 4, positive reviews accounted for 57.01% of the C level with no reviews. Negative reviews accounted for 43.27% of patients at the A level with reviews of more than 51 words. Therefore, it can be inferred that patients are willing to spend more time describing the course of events for negative reviews than for positive reviews. This also suggests that the negative reputation resulting from a service failure will significantly impact business performance.

**Table 4:** Number and percentage of positive and negative reviews’ importance levels

Rating	Rating description	PN	PN%	NN	NN%	TN	TN %
A	Reviews more than 51 words	23	10.41%	74	43.27%	97	24.74%
B	Reviews 1 ~ 50 words	72	32.58%	67	39.18%	139	35.46%
C	Only ratings, no reviews	126	57.01%	30	17.54%	156	39.80%
Total		221	100.00%	171	100.00%	392	100.00%

1. PN: number of positive reviews; NN: number of negative reviews; TN: total number of reviews

2. Positive review deduction (C) The number of samples that left a score but did not leave a message was 95(A + B). Negative review deduction (C) The number of samples that left a score but did not leave a message was 141(A + B)

### Comparative Analysis of the Number and Percentage of Positive and Negative Evaluations on the Five Dimensions of Service Quality

According to Table 5, “assurance” accounted for 60.00% of positive reviews, followed by “reliability” with 42.11%. “Assurance” accounted for 72.34% of negative reviews, followed by “responsiveness” with 28.37% and “reliability” with 26.95%. Overall, the two most important aspects that affect total positive and negative reviews are “assurance” at 67.37% and “reliability” at 33.05%. Therefore, it can be inferred that the most important factors that affect hospital service quality reviews are the professional skills and attitudes of medical staff and whether the patient’s medical issues can be solved. Service waiting is a factor that cannot be ignored in negative reviews.

**Table 5:** The number and percentage of positive and negative reviews on SERVQUAL’s five dimensions

Dimension	PN	PN% (N = 95)	NN	NN % (N = 141)	TN	TN % (N = 236)
Tangibles	25	26.32%	26	18.44%	51	21.61%
Reliability	40	42.11%	38	26.95%	78	33.05%
Responsiveness	6	6.32%	40	28.37%	46	19.49%
Assurance	57	60.00%	102	72.34%	159	67.37%
Empathy	9	9.47%	27	19.15%	36	15.25%

1. PN: number of positive reviews; NN: number of negative reviews; TN: total number of reviews

2. Each review may cover more than one aspect

3. The number of samples with positive reviews deducted without leaving a message was 95

4. The number of samples with negative reviews deducted without leaving a message was 141

### Comparative analysis of positive and negative reviews on the SERVQUAL Scale’s five dimensions

According to Table 6, the positive and negative reviews of the tangibles dimension were the highest, with “A4 Whether the hospital’s medical facilities are complete” (positive reviews 64.00%/negative reviews 42.31%), followed by “A1 Whether the public facilities of the hospital are complete” (positive rating 44.00%/negative rating 30.77%). This highlights the importance of the hospital’s medical facilities and public facilities.

**Table 6:** Number and percentage of positive and negative reviews on SERVQUAL’s five dimensions detailed questions

Dimension	Items	PN	PN %	NN	NN%	TN	TN%
Tangibles (PN = 25/NN = 26/TN = 51)	A1 Whether the public facilities of the hospital are complete	11	44.00%	8	30.77%	19	37.25%
	A2 Whether the barrier-free facilities of the hospital are complete	1	4.00%	1	3.85%	2	3.92%
	A3 Whether the hospital environment is clean, hygienic, and beautiful	4	16.00%	5	19.23%	9	17.65%
	A4 Whether the hospital’s medical facilities are complete	16	64.00%	11	42.31%	27	52.94%
	A5 Whether the clothing and appearance of medical staff are appropriate	0	0.00%	2	7.69%	2	3.92%
	A6 Whether the other tangible services of the hospital are perfect	1	4.00%	6	23.08%	7	13.73%
Reliability (PN = 40/NN = 38/TN = 78)	B1 Whether the medical staff can effectively treat the condition	28	70.00%	24	63.16%	52	66.67%
	B2 Whether the doctor can provide information of the condition and treatment in detail	14	35.00%	6	15.79%	20	25.64%
	B3 Whether medical staff provide medical services promptly	1	2.50%	10	26.32%	11	14.10%
	B4 Other reliability issues	1	2.50%	2	5.26%	3	3.85%
Responsiveness (PN = 6/NN = 40/TN = 46)	C1 Whether medical staff can address service requests quickly	4	66.67%	29	72.50%	33	71.74%
	C2 Whether medical staff can provide information about the service process and wait time	1	16.67%	18	45.00%	19	41.30%
	C3 Whether medical staff actively and willingly assist the patient	3	50.00%	6	15.00%	9	19.57%
	C4 Other responsiveness issues	0	0.00%	2	5.00%	2	4.35%
Assurance (PN = 57/NN = 102/TN = 159)	D1 Whether medical staff possess professional skills and knowledge	25	43.86%	35	34.31%	60	37.74%
	D2 Whether medical staff possess professional service communication attitude and etiquette	39	68.42%	74	72.55%	113	71.07%
	D3 Whether medical staff can provide medical services promptly	0	0.00%	1	0.98%	1	0.63%
	D4 Other assurance issues	0	0.00%	9	8.82%	9	5.66%
Empathy (PN = 9/NN = 27/TN = 36)	E1 Whether medical staff can meet individualized service demands	8	88.89%	11	40.74%	19	52.78%
	E2 Whether the hospital’s consultation time can provide multiple time options	0	0.00%	0	0.00%	0	0.00%
	E3 Whether the hospital can pay attention to the personal privacy of patients	0	0.00%	1	3.70%	1	2.78%
	E4 Other empathy issues	4	44.44%	12	44.44%	16	44.44%

(1) *PN* number of positive reviews, (2) *NN* number of negative reviews, (3) *TN* Total number of reviews

The reliability dimension had the highest number of positive and negative reviews, with “B1 Whether the medical staff can effectively treat the condition” (positive reviews: 70.00%; negative reviews: 63.16%). Positive reviews were followed by “B2 Whether the doctor can provide information about the condition and treatment in detail,” and negative reviews were followed by “B3 Whether medical staff provides medical services promptly.”

The responsiveness dimension was the highest in both positive and negative ratings: “C1 Whether medical staff can address service requests quickly” (positive reviews 66.67%/negative reviews 72.50%). Positive reviews were followed by “C3 Whether the medical staff actively and willingly assist the patient” (50.00%); negative reviews were followed by “C2 Whether the medical staff can provide information about the service process and wait time” (45.00%).

In the assurance dimension, the highest positive and negative reviews were “D2 Whether medical staff possesses professional service communication attitude and etiquette” (positively rated 68.42%/negatively rated 72.55%); the second-highest was “D1 Whether the medical staff possesses professional skills and knowledge” (positively rated 43.86%/negatively rated 34.31%). This highlights the importance of the professional attitude and skills of the hospital’s medical staff.

The empathy dimension was the highest rated: “E1 Whether medical staff can meet individualized service demands” (88.89% positive), followed by “E4 Other empathy issues” (44.44%). In contrast, “E4 Other empathy issues” (44.44%) had the highest number of negative reviews, followed by “E1 Whether medical staff can meet individualized service demands” (40.74%).

## Discussion

This study found 430 eWOM reviews of a hospital in Taiwan from June 24, 2011, to December 31, 2021. The overall average score was 3.135 points (out of 5), which is low. After screening, 38 comments that were not relevant to service quality were eliminated, and 221 positive eWOM and 171 negative eWOM comments were obtained. The statistical results found a sharp increase in eWOM reviews since 2015. Since the onset of COVID-19 in 2020, the growth of negative reviews has been larger than the number of positive reviews. The government’s strict health and welfare policies and uncontrollable factors in the medical environment (hospital crowd control, order of vaccine delivery, etc.) during the pandemic have caused the rapid increase in negative eWOM reviews. In the past, scholars believed that word-of-mouth reviews on websites were more positive than negative [24–26, 64–67]; however, this study found that since the outbreak of COVID-19 in 2020, the number of negative reviews has grown more than the number of positive reviews, showing that the outbreak has impacted hospitals’ service comments. The main reasons may be the government’s strict health and welfare policies during the epidemic as well as uncontrollable factors in the medical environment (crowd control in hospitals, vaccine delivery orders, etc.), resulting in a rapid increase in negative eWOM comments.

The results of this study are the same as those of previous studies. The valence of eWOM reviews is generally more positive than negative, but the COVID-19 epidemic has caused more negative than positive reviews. In addition, negative reviews are more influential than positive reviews, and there is a phenomenon of negativity bias [24–26, 64, 68–75].

In addition, the negative score was only 1 point, which accounted for 79.53%. A-level customers with a negative score of more than 51 words accounted for 43.27%, a very high proportion, indicating that patients are aware of and concerned about the hospital’s service negligence. The higher the number of negative comments, the lower the online score, similar to previous research [5, 60–63]. It is suggested that the hospital conduct a case analysis of these major service failures and propose effective countermeasures for improvement. Only in this way can the same service failures be prevented from recurring in the future. It is necessary to avoid the continuous expansion of a major negative reputation, which will affect the operational performance of the hospital.

In this study, semantic content analysis was used to classify the reviews according to the revised PZB SERVQUAL scale, and then statistical analysis was performed. The results of the study found that the hospital’s service quality evaluations, both positive and negative, performed most prominently in the “assurance” dimension of the SERVQUAL scale (positive rating of 60.00%/negative rating of 72.34%). “D2 Whether medical staff possesses professional service communication attitudes and etiquette” and “D1 Whether the medical staff possesses professional skills and knowledge” received the most attention in the detailed aspects. The results of this study highlight that “assurance” is the service quality item that patients are most likely to perceive in the five dimensions of service quality, and the professional attitude and courtesy of medical staff are more important than their professional skills. Dopeykar et al. pointed out - in order to improve patients’ satisfaction and improve service quality, clinic managers in the study should try to make patients aware of the staff’s expertise and competence, thereby increasing patients’ trust in staff and doctors. In addition, respecting the patient’s privacy, showing a friendly attitude and respectful behavior towards them, and explaining medical conditions and illnesses to the patient, can increase reassurance and their satisfaction, and ultimately the quality of service provided. The results of such a study are very similar to the results of this study [76].

The second dimension that affects positive word of mouth is “reliability (42.11%),” and the item “B1 Whether the medical staff can effectively treat the condition (70%)” is the highest. The third dimension that affects positive word of mouth is “tangibles (26.32%),” and item “A4 Whether the hospital’s medical facilities are complete (64%)” is the highest. This research result indicates that good medical effects and perfect medical equipment are easy ways to gain a positive reputation from patients. Ko and Chou pointed out - proper response to the demands of the resident population is crucial, as a professional, friendly, welcoming attitude can make the resident feel the staff is reliable and increase the resident’s sense of trust and safety; these service gaps Possibly related to medical staff training, experience, empathy, and understanding of the psychological needs of residents, skills in interacting with older adults can be improved through on-the-job training and experience sharing, resulting in higher levels of competence. The results of such a study are similar to the conclusions of this study [77].

The second most important dimension of negative word-of-mouth is “responsiveness (28.37%).” The subitem indicator “C1 Can medical staff quickly resolve service requests (66.67%)” is the highest. The third most important dimension of negative word-of-mouth is the “reliability (26.95%)” dimension, and the subitem “B1 can medical staff effectively treat the disease (70.00%)” is the highest. This study shows that poor “responsiveness” in the service process is more important than poor “reliability” for negative word of mouth. The occurrence is obvious. Therefore, medical institutions should improve service waiting problems in the medical service process, reduce patients’ pain while waiting for service, improve service efficiency and avoid a potentially negative reputation. According to Teshnizi et al. who compiled the literature related to the quality of medical services in the past, using the SERVQUAL questionnaire to evaluate service quality, in general, assurance and reliability present the largest service gaps (the so-called service gap refers to the gap between the customer’s perception and expectations calculated as the perception score minus the expectation score) among the five dimensions; tangible and empathy have smaller service gaps. It is shown that medical services are more difficult to meet customer expectations in terms of assurance and reliability than in tangible and empathy; this is similar to the conclusion of this study [50]. However, this study also found that eWOM’s immediate response is more limited by the performance of the professional skills and attitudes of current medical staff [assurance] and whether it can respond to customers’ service needs [responsibility] in a timely manner. The formation of negative eWOM is more important. This is also the greatest value of this study. The results of the SERVQUAL questionnaire survey, which was used in post-event recall in the past, were compared with the combination of immediate response positive and negative eWOM and SERVQUAL in this study, showing differences in the performance of five aspects of service quality.

## Conclusion

The results of this study show that the COVID-19 epidemic has changed the growth of positive and negative word of mouth, and negative eWOM has shown greater growth than positive eWOM. The uncertainty of the COVID-19 epidemic has impacted the service quality of the hospital. The hospital is facing an epidemic, and maintaining service quality in this ever-changing environment is the greatest challenge for current hospital operations and management. The most important finding of this study is that for both positive and negative word of mouth, the “assurance” dimension of the SERVQUAL scale is the most important. Compared to the findings of Nemati et al., hospitals handling COVID-19 patients need to pay special attention to ensure responsiveness to improve their service quality and manage nursing during the pandemic [78]. This also highlights the difference between word-of-mouth information and the SERVQUAL questionnaire.

The professional attitude and professional skills of medical staff determine the hospital’s word of mouth. Good medical effects in the “reliability” dimension and perfect medical equipment in the “tangibles” dimension will effectively improve the reputation of the hospital. The problem of service waiting in the “responsiveness” dimension and the poor medical effect in the “reliability” dimension are obvious sources of negative word of mouth. These are areas in which the hospital urgently needs to seek effective ways to improve. Therefore, this study recommends that the hospital strengthen the education and training of medical staff with regard to service contact and audit their working attitude. The hospital should seek to enhance the professional attitude and professional skills of medical staff and effectively solve the medical problems of patients. Finally, it is recommended that the hospital develop an integrated medical auxiliary service information system. Improving the information transparency and real-time response of the service process can reduce the pain of service waiting. This may improve positive WOM and reduce the eWOM effect of negative service reviews.

For future research, the following proposed problems should be addressed in conjunction with other decisions.

1. This study was conducted in a single hospital. Therefore, its explanatory power for all hospitals is slightly insufficient. It is recommended to conduct cross-regional or even cross-border related research comparisons on hospitals at different levels in the future, which can greatly improve the explanatory power of the inferences.
2. The single hospital eWOM targeted in this study was collected via Google Maps. A comparison of the word-of-mouth performance of the same institution on different social media is recommended.
3. This study summarizes the eWOM of a single hospital into SERVQUAL for analysis. Future research could summarize it into other service theoretical models (for example, HEALTHQUAL) for comparative analysis.
4. With the rapid development of big data AI analysis tools, it is suggested that large research and statistical software companies can develop statistical tools for eWOM semantic analysis in different languages, reduce the time-consuming and labor-intensive manual coding costs and subjective factors, and expand the scope of research to improve the quality and quantity of relevant research.

## Data Availability

The datasets used and analyzed during the current study are available from the corresponding author on reasonable request.
